# Supplementary material for: Facile Synthesis of Hollow Fe3O4-rGO Nanocomposites for the Electrochemical Detection of Acetaminophen
Source: Nanomaterials (Basel). 2023 Feb 12;13(4):707. doi: 10.3390/nano13040707 (PMC9964092; doi:10.3390/nano13040707)
Supplement: Supplementary file 1 [file nanomaterials-13-00707-s001.zip › nanomaterials-2178319-supplementary.pdf]

Table S1 Comparison of analytical performance of AC detection limit by different modified electrodes.

Modified electrodes	Techniques	Detection limit/μM	Reference
$Cd(OH)_2$-rGO/GCE	DPV	0.08	[3]
AuNP-PGA/SWCNT	DPV	1.18	[11]
AuNPs/$SiW_{11}Cu$/MWCNTs/GCE	DPV	0.42	[12]
MCNs/GCE	DPV	0.34	[13]
CNNS/GO/GCE	DPV	0.017	[14]
phosphorus-doped graphene/GCE	DPV	0.36	1
electrospun CeBiOx nanofibers/SPE	DPV	0.2	2
(CDA)/Au-AgNP	CV	2.6	3
β-CD-RGO	CV	2.3	4
MWCNT-polymer/GCE	CV	3.5	5
Fe_3O_4-rGO/GCE	SWV	0.11	This work

1. Zhang, X.; Wang, K.P.; Zhang, L.N.; Zhang, Y.C.; Shen, L. Phosphorus-doped graphene-based electrochemical sensor for sensitive detection of acetaminophen. *Anal. Chim. Acta* 2018, 1036, 26-32.
2. Cao, F.; Dong, Q.; Li, C.; Chen, J.; Ma, X.; Huang, Y.; Song, D.; Ji, C.; Lei, Y. Electrochemical sensor for detecting pain reliever/fever reducer drug acetaminophen based on electrospun CeBiO nanofibers modified screen-printed electrode. *Sens. Actuators B Chem.* 2018, 256, 143-150.
3. Wei, R. Biosynthesis of Au-Ag alloy nanoparticles for sensitive electrochemical determination of paracetamol. *Int. J. Electrochem. Sci.* 2017, 12, 9131-9140.
4. Fu, L.; Lai, G.; Yu, A. Preparation of β-cyclodextrin functionalized reduced graphene oxide: application for electrochemical determination of paracetamol. *RSC Adv.* 2015, 5, 76973-76978.
5. Liu, R.; Zeng, X.; Liu, J.; Luo, J.; Zheng, Y.; Liu, X. A glassy carbon electrode modified with an amphiphilic, electroactive and photosensitive polymer and with multi-walled carbon nanotubes for simultaneous determination of dopamine and paracetamol. *Microchim. Acta* 2016, 183, 1543-1551.
